# Case Report: A case of infantile acute hyperleukocytic leukemia treated by leukapheresis

**DOI:** 10.3389/fped.2024.1497943

**Published:** 2024-12-09

**Authors:** Shuang Bian, Nannan Li, Hui Gao

**Affiliations:** Department of Hematology and Oncology, Dalian Women and Children’s Medical Group, Dalian, China

**Keywords:** leukapheresis, acute hyperleukocytic leukemia, infant, acute lymphoblastic leukemia, white blood cells

## Abstract

Leukapheresis is a treatment used to reduce leukocytes to decrease the number of white blood cells in circulation and prevent the risks of hyperviscosity and cerebrovascular and pulmonary leukostasis. We present a case of pro-B-cell acute lymphoblastic leukemia (ALL) with hyperleukocytosis in a 6-month-old infant, characterized by a positive KMT2A/AFF1 fusion gene with a leukocyte count of 1,755 × 10^9^/L. After two consecutive sessions of leukapheresis, the white blood cell count decreased to 55 × 10^9^/L. The infant recovered after the high-dose methotrexate chemotherapy. These results demonstrate that leukapheresis is a feasible treatment option for acute hyperleukocytic leukemia in infants with ALL.

## 1 Introduction

Acute hyperleukocytic leukemia is defined as acute leukemia (AL) with an initial total blood cell or blast count higher than 100,000/µl (1, 2). Hyperleukocytosis can lead to early morbidity and mortality as a result of leukemic cell proliferation (3, 4). Approximately 10%–20% of children who are newly diagnosed with AL present with hyperleukocytosis and are at risk for acute respiratory distress syndrome, intracranial hemorrhage, and stroke (5, 6). Complications such as leukostasis, tumor lysis syndrome, and disseminated intravascular coagulation, rather than high leukocytosis count, put the patients at risk and require therapeutic intervention (7, 8).

The rapid mechanical removal of excess leukocytes through leukapheresis has become a routine procedure in many hematology centers (9, 10). A single leukapheresis procedure can reduce the white blood cell (WBC) count by 10%–70%. Multiple leukapheresis procedures are often necessary to effectively decrease the burden of leukemic cells (9). However, the date on the application of leukapheresis in the treatment of acute hyperleukocytic leukemia is limited (11), and the efficacy of leukapheresis in children, especially in low-weight infants, remains controversial. Here, we describe the successful treatment of an approximately 6-month-old infant with acute lymphoblastic leukemia (ALL) at risk of hyperleukocytosis.

## 2 Case description

A 6-month-old Asian male infant presented with paroxysmal coughing without obvious inducement and difficulty in expectorating sputum for 1 week. The day before admission, the infant experienced wheezing that worsened after physical activity, but there were no apparent signs of dyspnea or asthma. His complete blood count (CBC) revealed an abnormal white blood cell (WBC) count of 1,560 × 10^9^/L (1,560,000/mm^3^) (Table 1). Laboratory tests showed hemoglobin (HGB) levels at 49 g/L and a platelet count (PLT) of 32 × 10^9^/L. Consequently, the infant was referred to our emergency room for further evaluation because of suspected leukemia.

**Table 1:** Clinical and biochemical characteristics in infantile patient.

Test	Preadmission	Admission	First postoperative	Second preoperative	Second postoperative	Normal value
WBC (×10^9^/L)	1,560	1,755	946.52	493.47	55.63	4.8–14.6
NEUT (%)	–	–	1	–	7.9	9–57
LYMPH (%)	–	–	82.4	–	74.9	31–81
MON (%)	–	–	16	–	17	2–13
EO (%)	–	–	0	–	0	1–10
BASO (%)	–	–	0.6	–	0.2	0–1
NEUT (×10^9^/L)	–	–	9.69	–	4.41	0.8–6.4
LYMPH (×10^9^/L)	–	–	779.63	–	41.66	2.5–9
MONO (×10^9^/L)	–	–	151.61	–	9.46	0.17–1.06
EO (×10^9^/L)	–	–	0.04	–	0.01	0.07–1.02
BASO (×10^9^/L)	–	–	5.55	0	0.09	0–0.1
RBC (×10^12^g/L)	2.09	1.79	2.36	2.46	2.13	4.0–5.5
HGB (g/L)	49	56	56	65	62	97–141
HCT (%)	23.4	13.7	23.9	23.5	18.4	30–41
MVC (fL)	112	76.1	101.3	95.3	86.4	72–85
MCHC (g/L)	209	407	234	277	337	311–355
MCH (pg)	23.4	31	23.7	26.4	29.1	24–30
RDW-CV (%)	12.7	12.7	–	25.9	24.3	10–20
RDW–SD (fL)	32	31.5	–	73	70.3	35–56
PLT(×10^9^/L)	–	31	24	89	48	190–579
PCT (%)	–	0.021	–	0.088	0.05	0.08–0.32
MPV (fL)	–	6.6	–	16.3	10.7	10–18
PDW (%)	–	15.7	–	9.8	13.1	6–10

WBC, white blood cell; NEUT (%), neutrophil ratio; LYMPH (%), lymphocyte ratio; MON (%), monocyte ratio; EO (%), eosinophil ratio; BASO (%), basophil ratio; NEUT, neutrophil count; LYMPH, lymphocyte count; MONO, monocyte count; EO, eosinophil count; BASO, basophil count; RBC, red blood cell; HGB, hemoglobin; HCT, hematocrit; MVC, mean corpuscular volume; MCHC, mean corpuscular hemoglobin concentration; MCH, mean corpusular hemoglobin; RDW, red blood cell volume distribution width; PLT, platelet count; PCT, plateletocrit; MPV, mean platelet volume; PDW, platelet distribution width.

Nebulization and intramuscular injection with 3 mg dexamethasone resulted in improved wheezing symptoms. Physical examination values were as follows: body temperature, 36.6℃; respiration, 44 beats/min; heart rate, 131 beats/min; blood oxygen saturation, 92%; blood pressure, 97/62 mmHG (1 mmHg = 0.133 kPa); alertness, general response, and slight shortness of breath; liver, 4 cm below the rib; and spleen, 5 cm below the rib, tough. After admission to our hospital pediatric intensive care unit, the patient underwent continuous positive airway pressure (CPAP, oxygen 2 empty 4), fasting and rehydration, ceftriaxone anti-infection, mannitol, furosemide, hydration and alkalinization, and allopurinol support symptomatic treatment. Over the next day, the WBC count stayed at 1,755 × 10^9^/L (1,755,000/mm^3^), and the differential showed a blast cell count of 94%. Other examination values included Hb 56 g/L and PLT 31 × 10^9^/L. We made a preliminary inference of hematological cancer and conducted five tests, including (1) bone marrow smear, bone marrow, and cerebrospinal fluid immune classification, (2) bone marrow genetic screening for 57 types of leukemia, (3) bone marrow chromosome karyotype analysis, (4) IG rearrangement sequencing of the immunized group in the bone marrow library, and (5) whole transcriptome sequencing.

**Figure 1:**
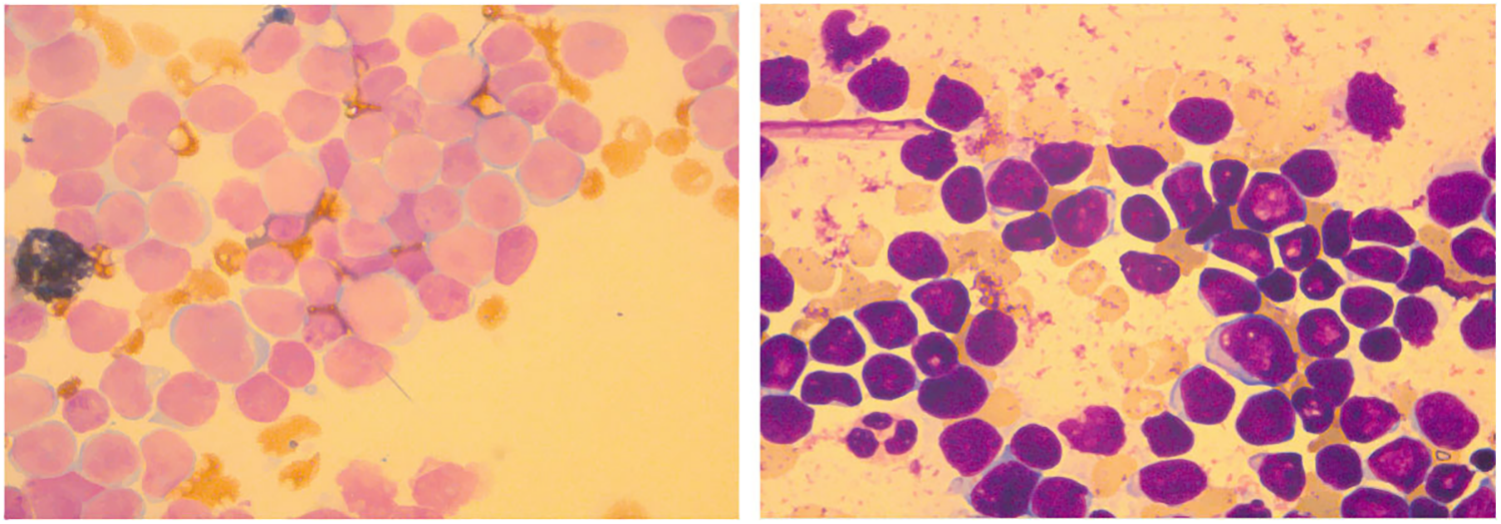
Results of the bone marrow smear.

Bone marrow was under a hyperactive condition, mainly manifested as a granulocyte: nucleated red blood cell (RBC) ratio of 3:1. It also was characterized by malignant proliferation of primitive cells. The primitive cells were different in size, mostly small and regular in appearance. The nuclei showed depression, distortion, and cleft changes with dense and thick chromatin. The amount of cytoplasm was small; no granules were seen and some vacuoles were seen. The bone marrow smear showed increased cell fragmentation, inhibited proliferation of red, granulocyte, giant cells and PLT, and negative POX expression (Figure 1). In terms of bone marrow immune classification, abnormal B primitive lymphocytes were detected in the bone marrow test, indicating a tendency towards the pro-B-ALL stage. The expression profile showed CD34 (+), CD19 (+), cCD79a (+), TDT (+), CD9 (+), HLA-DR (+), CD123 (+), CD38 (+), CD7 (−), CD2 (−), MPO (−), CD56 (−), CD4 (−), CD10 (−), CD99 (−), CD64 (−), CD22 (−), CD117 (−), CD13 (−), CD20 (−), CD33 (−), CD96 (−), CD61 (−), CD8 (−), kappa (−), lambda (−), and IgM (−) (Supplementary Table S1). The same result was also observed in abnormal B lymphocytes, which accounted for 21.16% of nuclear cells expressing CD19 (+), CD38 (+), and CD34 (+) in cerebrospinal fluid (Supplementary Table S2).

**Figure 2:**
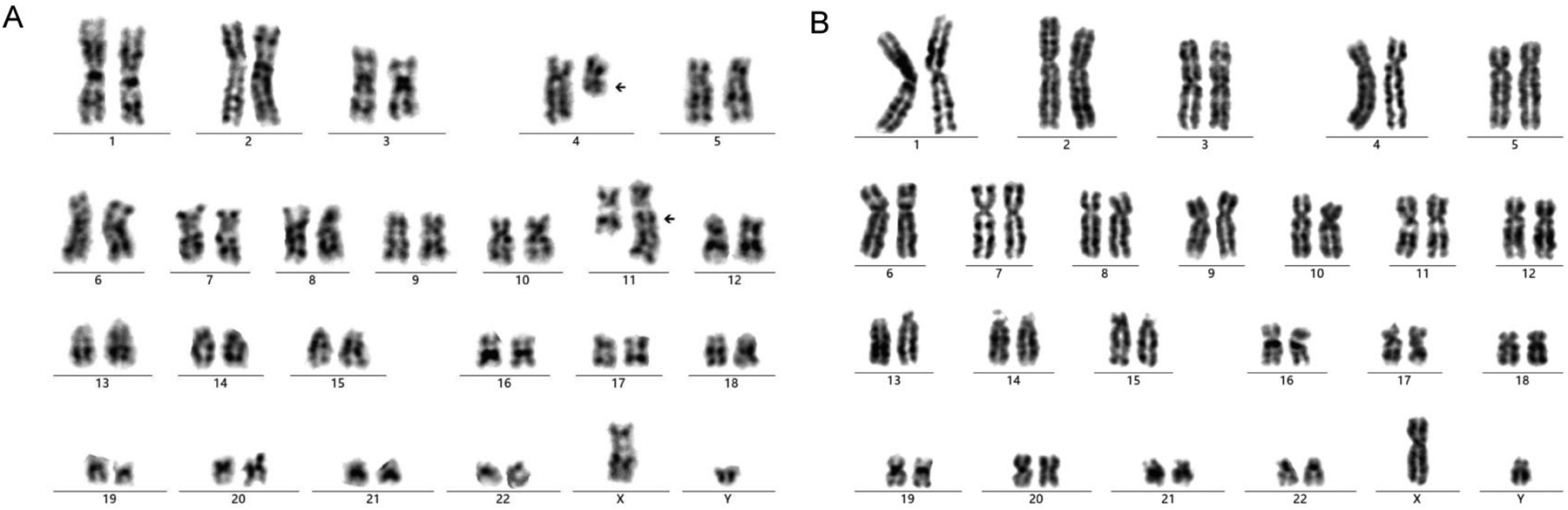
The result of bone marrow chromosome karyotype analysis in the admission **(A)** and post-treatment **(B)**.

Genetic screening of bone marrow for 57 types of leukemia identified a positive KMT2A/AFF1 fusion gene (Supplementary Table S3). Bone marrow karyotype analysis revealed 46, XY, t (4; 11) (q21; q23) (7). Analysis of six metaphase division phases revealed reciprocal translocations of chromosomes 4 and 11 (Figure 2A). IG rearrangement by immune repertoire sequencing using multiplex PCR combined with next-generation sequencing technology did not identify any master clonal sequence involving IGH, IGK, or IGL gene rearrangements (Supplementary Figure S1). Whole transcriptome sequencing results indicated the presence of KMT2A/AFF1 fusion gene (grade 1) as well as the detection of an AFF1/KMT2A fusion gene (grade 3) (Supplementary Table S4 and S5; Figure S2). The KMT2A/AFF1 fusion gene is mainly found in pre-infantile B-ALL patients. These findings confirmed a diagnosis of pro-B-cell ALL with positive outcomes for the KMT2A/AFF1 fusion gene.

Because of a decrease in transcutaneous oxygen protection to 75%–78% with sudden dyspnea, the infant was intubated and assisted by a ventilator (mode: PC/AC; parameters: respiratory rate, 25/min; timed inspiration, 0.7 s; pressure support ventilation, 12 cm H_2_O; positive end-expiratory pressure, 10.0 cm H_2_O; fraction of inspiration O_2_, 80%). Fentanyl and midazolam were given for analgesia and sedation. Physical examination showed unconsciousness; irregular breathing with the three concave signs; pale and cyanotic around the mouth; no rash, no bleeding spots, and no marble patterns on the whole body; the superficial lymph nodes were not palpable and enlarged; bilateral pupils were equally large, perfectly round, with a diameter of 3.0 mm; symmetrical chest, thick respiratory sounds on auscultation of both lungs, scattered phlegm wheezing sounds; the abdomen was not swollen and the texture is soft; enlargement of the liver and spleen below the navel; and limb peripheral temperature CRT2S.

After thorough discussion and consultation with the patient's family, the infant underwent 70 min of leukapheresis in the first session. Leukapheresis was performed using the auto MNC procedure employed on COM.TEC Fresenius blood component separator (Fresenius SE & Co. KGaA, Germany) with the placement of a 6.5 F double-chamber hemofiltration intravenous catheter was placed in the femoral center. Because of the diagnosis of pro-B-cell ALL and to prevent tumor lysis, we initiated remission induction chemotherapy with low-dose dexamethasone 1.5 mg (3.2 mg/m^2^). The results of the blood count examination revealed WBC, 946.52 × 10^9^/L (946,520/mm^3^); Hb, 56 g/L; and PLT, 24 × 10^9^/L (Table 1).

On the third day of admission, the infant had a sudden fever with a maximum temperature of 38.5℃, and ibuprofen was given by nasal feeding to reduce fever. During the procedure, the infant showed one convulsion, which manifested as an airway spasm, peak pressure of the ventilator, slightly stiff limbs, and slightly upturned eyes. We administered midazolam, propofol, and rocuronium for temporary use, and mannitol and glycerin fructose were given to reduce intracranial pressure, and the seizures were relieved. To ensure the smooth progress of the second round of leukapheresis, we conducted CBC tests. The results showed WBC, 493.47 × 10^9^/L (4,934,700/mm^3^); Hb, 65 g/L; and PLT, 89 × 10^9^/L (Table 1). Leukapheresis was performed successfully with stability intraoperative monitoring and maintenance of blood electrolytes for 100 min. Immediately after the procedure, we administered an intravenous infusion of adequate dexamethasone 2.7 mg (6 mg/m^2^) and checked the blood cell count. The CBC showed WBC, 55.63 × 10^9^/L; Hb, 62 g/L; and PLT, 89 × 10^9^/L (Table 1). One week after leukapheresis, the infant was doing well after being weaned from mechanical ventilation on the third postoperative day and transferred to the hematology and oncology ward on the fifth postoperative day.

After nearly half a year of high-dose methotrexate chemotherapy treatment according to the Chinese Children Cancer Group Infant Acute Lymphoblastic Leukemia 2022 collaborative group protocol (CCCG-iALL-2022) (12, 13) (Supplementary Table S8), the child was successfully given to significantly, alter minimal residual disease (leukemia residue cells) for lower than 10^−4^ (Supplementary Figure S3; Table S6) and the KMT2A/AFF1 fusion gene for 0 (Supplementary Table S7). Bone marrow karyotype analysis again revealed no numerical or structural chromosomal abnormalities associated with the tumor with 46, XY (Figure 2B). Currently, the child, at 1 year and 1 month old, shows an excellent prognosis for recovery with stable vital signs and similar growth and development as infants at the same age. The CBC and biochemical indicators are within the normal range. Leukapheresis was approved by Dalian Medical and Health New Technology Application (number: 2021-37). The patient's guardian signed the informed consent form (DLET-JS-2021-07).

## 3 Discussion

In patients with AL, leukostasis secondary to hyperleukocytosis is a serious and life-threatening complication, indicating a poor prognosis (14, 15). Leukapheresis is considered an emergency life-saving procedure (9), capable of effectively reducing the abnormally elevated WBC count in leukemia patients, thereby alleviating related symptoms and enhancing prognosis. Leukapheresis is typically considered when certain symptoms manifest, such as in patients with ALL when the WBC count in the peripheral blood is 400 × 10^9^/L and in those with acute myeloid leukemia presenting with a WBC count of >100 × 10^9^/L (16, 17). The latest guideline of the American Apheresis Therapy Society defines leukemia with a peripheral WBC count of >100 × 10^9^/L as hyperleukocytic leukemia and recommends leukostasis caused by abnormally increased WBC in patients with hyperleukocytic leukemia as a class I indication for therapeutic leukapheresis (4). However, a number of recent reports, including an international multicenter retrospective study, showed that the evidence supporting its benefit in extremely young patients with hyperleukocytosis is still insufficient (18–20). We present this case to contribute to the existing evidence for the treatment effectiveness of leukapheresis for hyperleukocytosis.

Leukapheresis is a technique used to reduce the concentration of leukocytes in the human body, aiming to manage or prevent hyperviscosity (14). There is an association of hyperleukocytosis with specific subtypes of ALL with association with t (4:11) (21) and t (9:22) (22). In the current case, the infant's WBC count was as high as 1,755 × 10^9^/ml upon admission, and the condition was critical with leukostasis. Because the infant was only approximately 6 months old, we carefully diagnosed infantile acute hyperleukocytic leukemia in the pro-B-ALL stage considering the corresponding results. We also explored various surgical options. Ultimately, we decided to proceed with leukapheresis as a foundation for chemotherapy.

Our team considers leukocytosis as an independent risk factor for early death. Leukocytosis itself increases blood viscosity. The adhesion of leukemic cells to capillary endothelium and activation of endothelial cells induced by cytokines further increased blood viscosity, causing blood stasis and life-threatening complications such as intracranial hemorrhage and respiratory distress, increasing the risk of early death of patients (14). Leukapheresis can rapidly reduce tumor cells and reduce early mortality, especially in this infant's condition with an extremely high number of WBC. Here, we performed two sessions of leukapheresis. The WBC count decreased dramatically to 55 × 10^9^/L, representing a decrease of 96%. This result demonstrated the reduction of WBC count to the safe range, and there were no complications related to tumor lysis. We considered not only the fatal consequences of hyperleukocytosis but also the risk of death from tumor lysis syndrome for the patient; therefore, we administered low-dose remission induction chemotherapy during the leukapheresis procedure.

Leukapheresis-associated complications mostly include neurological and respiratory issues, such as hypocalcemia, catheter dysfunction, coagulopathy, and catheter-related thrombosis (18, 23). Notably, the current patient did not experience any complications. This may be attributed to our meticulous therapeutic manipulation throughout the procedure for the infant (24). Precise contemporary supportive care combined with prompt diagnosis and initiation of appropriate chemotherapy can effectively reduce fatalities associated with leukocytosis-induced complications. Our team also expressed significant concern regarding the hemoglobin and platelet laboratory test results and promptly ensured the availability of platelet follow-up for thrombocytopenia (22). The early and proactive reduction of white blood cells, coupled with subsequent attenuation in blood viscosity, plays a pivotal role in facilitating chemotherapy administration to patients. The infant's white blood cell count was effectively regulated within normal levels following methotrexate chemotherapy, demonstrating the effectiveness of the treatment. After a period of observation and monitoring, the patient's clinical symptoms were significantly improved and all vital signs were stable, allowing the infant's safe discharge with their family. All the procedures not only reflect the accurate evaluation and management of the infant's conditions but also reflect the important role of leukapheresis in the treatment of acute hyperleukocytic leukemia. This case provides new evidence that infants at a young age can benefit from leukapheresis treatment and indicates similar treatment plans for similar cases in the future.

## Data Availability

The original contributions presented in the study are included in the article/Supplementary Material, further inquiries can be directed to the corresponding author.

## References

[1] Ali AM Mirrakhimov AE Abboud CN Cashen AF. Leukostasis in adult acute hyperleukocytic leukemia: a clinician’s digest. Hematol Oncol. (2016) 34:69–78. 10.1002/hon.2292 27018197

[2] Lafarge A Chean D Whiting L Clere-Jehl R. Groupe de Recherche en Réanimation respiratoire du patient d’Onco-Hématologie (GRRR-OH), clinical research in intensive care and sepsis—TRIal group for global evaluation and research in SEPsis (CRICS-TRIGGERSEP). Management of hematological patients requiring emergency chemotherapy in the intensive care unit. Intensive Care Med. (2024) 50:849–60. 10.1007/s00134-024-07454-z 38748265 PMC11164740

[3] Röllig C Ehninger G. How I treat hyperleukocytosis in acute myeloid leukemia. Blood. (2015) 125:3246–52. 10.1182/blood-2014-10-551507 25778528

[4] Padmanabhan A Connelly-Smith L Aqui N Balogun RA Klingel R Meyer E Guidelines on the use of therapeutic apheresis in clinical practice—evidence-based approach from the writing committee of the American Society for Apheresis: the eighth special issue. J Clin Apher. (2019) 34:171–354. 10.1002/jca.21705 31180581

[5] Eguiguren JM Schell MJ Crist WM Kunkel K Rivera GK. Complications and outcome in childhood acute lymphoblastic leukemia with hyperleukocytosis. Blood. (1992) 79:871–5. 10.1182/blood.V79.4.871.bloodjournal794871 1737097

[6] Park KM Yang EJ Lee JM Hah JO Park SK Park ES Treatment outcome in pediatric acute lymphoblastic leukemia with hyperleukocytosis in the Yeungnam region of Korea: a multicenter retrospective study. J Pediatr Hematol Oncol. (2020) 42:275–80. 10.1097/MPH.0000000000001771 32134842

[7] Bunin NJ Pui CH. Differing complications of hyperleukocytosis in children with acute lymphoblastic or acute nonlymphoblastic leukemia. J Clin Oncol. (1985) 3:1590–5. 10.1200/JCO.1985.3.12.1590 3864942

[8] Kittivisuit S Jongthitinon N Sripornsawan P Songthawee N Chavananon S Limratchapong C Hyperleukocytosis in childhood acute leukemia: early complications and survival outcomes. Cancers (Basel). (2023) 15:3072. 10.3390/cancers15123072 37370683 PMC10295972

[9] Zhang D Zhu Y Jin Y Kaweme NM Dong Y. Leukapheresis and hyperleukocytosis, past and future. Int J Gen Med. (2021) 14:3457–67. 10.2147/IJGM.S321787 34285568 PMC8286901

[10] Nguyen R Jeha S Zhou Y Cao X Cheng C Bhojwani D The role of leukapheresis in the current management of hyperleukocytosis in newly diagnosed childhood acute lymphoblastic leukemia. Pediatr Blood Cancer. (2016) 63:1546–51. 10.1002/pbc.26056 27187265 PMC5131872

[11] Woloskie S Armelagos H Meade JM Haas D. Leukodepletion for acute lymphocytic leukemia in a three-week-old infant. J Clin Apher. (2001) 16:31–2. 10.1002/jca.1006 11309829

[12] Chinese Children’s Cancer Group Acute Lymphoblastic Leukemia 2015 Study Group. Report of Chinese children’s cancer group acute lymphoblastic leukemia 2015 multicenter study. Zhonghua Er Ke Za Zhi. (2022) 60:1002–10. 10.3760/cma.j.cn112140-20220719-00895 36207846

[13] Leung AWK Cai J Wan Z Qin J Fang Y Sun L Outcome of infants with acute lymphoblastic leukemia treated with the Chinese Children's Cancer Group Acute Lymphoblastic Leukemia 2015 study protocol. Haematologica. (2024) 109:2726–31. 10.3324/haematol.2024.285201 38634141 PMC11290514

[14] Liu Y Issah MA Hu X Shen J. Fatal hyperleukocytic T lymphoblastic lymphoma/leukemia complicated with multi-gene fusion and mutation: clinical revelation and perception. Am J Blood Res. (2020) 10:440–6. 33489453 PMC7811898

[15] Ruggiero A Rizzo D Amato M Riccardi R. Management of hyperleukocytosis. Curr Treat Options Oncol. (2016) 17:7. 10.1007/s11864-015-0387-8 26820286

[16] Aqui N O'Doherty U. Leukocytapheresis for the treatment of hyperleukocytosis secondary to acute leukemia. Hematology Am Soc Hematol Educ Program. (2014) 2014:457–60. 10.1182/asheducation-2014.1.457 25696894

[17] Ganzel C Becker J Mintz PD Lazarus HM Rowe JM. Hyperleukocytosis, leukostasis and leukapheresis: practice management. Blood Rev. (2012) 26:117–22. 10.1016/j.blre.2012.01.003 22364832

[18] Stahl M Shallis RM Wei W Montesinos P Lengline E Neukirchen J Management of hyperleukocytosis and impact of leukapheresis among patients with acute myeloid leukemia (AML) on short- and long-term clinical outcomes: a large, retrospective, multicenter, international study. Leukemia. (2020) 34:3149–60. 10.1038/s41375-020-0783-3 32132655 PMC8155811

[19] Korkmaz S. The management of hyperleukocytosis in 2017: do we still need leukapheresis? Transfus Apher Sci. (2018) 57:4–7. 10.1016/j.transci.2018.02.006 29477941

[20] Bewersdorf JP Giri S Tallman MS Zeidan AM Stahl M. Leukapheresis for the management of hyperleukocytosis in acute myeloid leukemia-A systematic review and meta-analysis. Transfusion. (2020) 60:2360–9. 10.1111/trf.15994 32776542 PMC8631180

[21] Johansson B Moorman AV Haas OA Watmore AE Cheung KL Swanton S Hematologic malignancies with t(4;11)(q21;q23)–a cytogenetic, morphologic, immunophenotypic and clinical study of 183 cases. European 11q23 workshop participants. Leukemia. (1998) 12:779–87. 10.1038/sj.leu.2401012 9593281

[22] Foà R Chiaretti S. Philadelphia Chromosome-Positive acute lymphoblastic leukemia. N Engl J Med. (2022) 386:2399–411. 10.1056/NEJMra2113347 35731654

[23] Abla O Angelini P Di Giuseppe G Kanani MF Lau W Hitzler J Early complications of hyperleukocytosis and leukapheresis in childhood acute leukemias. J Pediatr Hematol Oncol. (2016) 38:111–7. 10.1097/MPH.0000000000000490 26794706

[24] Yilmaz D Karapinar B Karadaş N Duyu M Yazici P Ay Y Leukapheresis in childhood acute leukemias: single-center experience. Pediatr Hematol Oncol. (2014) 31:318–26. 10.3109/08880018.2013.818747 23988130

