# Supplementary material for: Case Report: A case of infantile acute hyperleukocytic leukemia treated by leukapheresis
Source: Front Pediatr. 2024 Dec 9;12:1497943. doi: 10.3389/fped.2024.1497943 (PMC11663633; doi:10.3389/fped.2024.1497943)
Supplement: Supplementary file 1 [file Table1.docx]

**Supplementary Table 1** Cell population proportion in the bone marrow blood tumor immune classification.

| Cell type | Result |
| --- | --- |
| Total Nucleated cell | 30000 |
| Abnormal naive B lymphocytes (%) | 93.03 |
| CD34 | + |
| CD19 | + |
| CD79a | + |
| TDT | + |
| CD9 | + |
| HLA-DR | + |
| CD123 | + |
| CD38 | + |
| CD7 | - |
| CD2 | - |
| MPO | - |
| CD56 | - |
| CD4 | - |
| CD10 | - |
| CD99 | - |
| CD64 | - |
| CD22 | - |
| CD117 | - |
| CD13 | - |
| CD20 | - |
| CD33 | - |
| CD96 | - |
| CD41a | - |
| CD16 | - |
| CD11b | - |
| CD36 | - |
| CD42a | - |
| CD3 | - |
| CD5 | - |
| CD300e | - |
| CD15 | - |
| CD14 | - |
| Kappa | - |
| Lambda | - |
| IgM | - |
| CD8 | - |

**Supplementary Table 2** Cell population proportion in cerebrospinal fluid immunophenotyping.

| Cell type | Number |
| --- | --- |
| Nucleated cell | 4924 |
| Abnormal naive B lymphocytes  (CD19^+^CD38^+^CD34^+^) | 1042 (21.16%) |

**Supplementary Table 3** The result of leukemia gene screening for 57 types of leukemia.

| **Test item** | **Result** |
| --- | --- |
| MLL-AF4 (KMT2A/AFF1) | + |
| MLL-AF6 | - |
| MLL-AF9 | - |
| MLL-AF10 | - |
| MLL-AF17 | - |
| MLL-AF1P | - |
| MLL-AF1Q | - |
| MLL-AFX | - |
| MLL-ELL | - |
| MLL-ENL | - |
| dupMLL | - |
| CBF β-MYH11 | - |
| AML1-ETO | - |
| AML1-MDS1 | - |
| SET-CAN | - |
| DEK-CAN | - |
| PML-RAR α | - |
| PLZF-RAR α | - |
| NPM-RAR α | - |
| STAT5b-RAR α | - |
| NuMA1-RAR α | - |
| PRKARIA-RAR a | - |
| FIPIL1-RAR α | - |
| NPM-ALK | - |
| ETV6-RUNX1(TEL-AML1) | - |
| TCF3-PBX1(E2A-PBX1) | - |
| BCR-ABL1 | - |
| NPM-MLF1 | - |
| TEL-ABL1 | - |
| TCF3-HLF(E2A-HLF) | - |
| TLS-ERG | - |
| SIL-TAL1 | - |
| ETV6-PDGFR α | - |
| FIPIL1-PDGFR α | - |
| ETV6-PDGFR β | - |
| NuP98-HOxA9 | - |
| NuP98-HOxA11 | - |
| NuP98-HOxA13 | - |
| NuP98-H0xC11 | - |
| NuP98-H0xD13 | - |
| NuP98-PMX1 | - |
| EVI1 | - |
| HOX11 | - |

**Supplementary Table 4** Blood tumor transcriptome sequencing gene locus.

| 5' gene | 3' gene | 5' gene breakpoint location | 3' gene breakpoint location | Fusion approach |
| --- | --- | --- | --- | --- |
| KMT2A | AFF1 | chr11:118355690: + | chr4:88011136: + | exonic/exonic |
| AFF1 | KMT2A | chr4:88005316: + | chr11:118359329: + | exonic/exonic |

**Supplementary Table 5** The result of blood tumor transcriptome sequencing in the diagnosis.

| Test | Result | Grade* |
| --- | --- | --- |
| KMT2A :: AFF1 fusion gene | + | 1 |
| AFF1 :: KMT2A fusion gene | + | 3 |

*Grade 1: mutations with clear clinical significance, including: level A, drug treatment or drug resistance targets approved by the National Food and Drug Administration (NMPA), the United States Food and Drug Administration (FDA) and other institutions, or mutations with clear diagnostic, therapeutic, and prognostic significance in the professional guidelines for the diagnosis and treatment of hematological malignancies; level B, mutations with diagnostic, therapeutic and prognostic significance in hematological malignancies based on expert consensus or authoritative literature.

Grade 2: mutations with potential clinical significance, including: level C, mutations with diagnostic, therapeutic or prognostic significance in hematological malignancies reported by several small-scale studies, but no consensus has been reached; level D, variant associated with a therapeutic target in preclinical trials or a newly identified somatic mutation in an important domain of a gene involved in the disease.

Grade 3: mutations of uncertain clinical significance, including low distribution frequency in the population, and no reported SNP of the patient itself

**Supplementary Table 6** Bone marrow tumor minimal residual disease detection of the proportion of various types of cells in the post-treatment.

| Cell type | Proportion (%) |
| --- | --- |
| Lymphocytes | 7.50 |
| CD34^+^CD10^+^CD19^+^ | 0.06 |
| CD34^-^CD10^+^CD19^+^ | 0.003 |
| Monocytes | 5.81 |
| Granulocytes | 73.09 |
| Primitive areas | 2.58 |
| Nucleated erythrocytes | 11.02 |
| Leukemia residue cells | <10^-4^ |

**Supplementary Table 7** The result of blood tumor transcriptome sequencing in the post-treatment.

| Test | Result |
| --- | --- |
| KMT2A :: AFF1 fusion gene | - |
| AFF1 :: KMT2A fusion gene | + |
| Negative control | - |
| Positive control | + |
| Internal control | - |

**Supplementary Table 8 Chemotherapy regimens**

| Stage | Name | Chemotherapy (dose/duration) |
| --- | --- | --- |
| I | Window period + induction remission period | Dexamethasone (MTX): oral/intravenous 6 mg/m^2^/day, Day 1-5, dexamethasone: oral/intravenous 8 mg/m^2^/day, Day 6-29 days, reduced and stopped for a week |
|  |  | Vincristine (VCR): 1.5 mg/m^2^, Day 6, 13, 20, 27 |
|  |  | Pegaspargase: 2000 U/m^2^, intramuscular injection, Day7 |
|  |  | Venetoclax (VEN): dose adjusted for age, Days 6-19 |
|  |  | Triple intrathecal injection: Day 6, 13, 20 |
| II | Treatment Period of berintoxime | Berintopol: 5 μg/m^2^/ day, 24 h IV infusion, Day 1-2, 15 μg/m^2^/ day, 24 h IV infusion, Day 3-16 |
|  |  | Triple sheath injection: Day 1, 8 |
| III | Early consolidation therapy (modified MARMA regimen) | High-dose methotrexate: 5000 mg/m^2^, IV (the first 10% of the dose was infused within 30 minutes, the remaining 90% within 23.5 hours) |
|  |  | Leucovorin rescue: 15 mg/m^2^, orally or intravenously, at 42, 48 and 54 hours after HD-MTX |
|  |  | Triple intrathecal injection: Day 1, 15 |
|  |  | Mercaptopurine (6-MP): 25 mg/m^2^/d, oral, Day 1-28 |
|  |  | High-dose cytarabine: 3000 mg/m^2^, IV, every 12 hours, Day 29-30 |
|  |  | PEG-Asp: 2000 U/m^2^, intramuscular injection, Day 31, 52 |
| IIII | Re-induction therapy | Dexamethasone: 8 mg/m^2^/ day, oral/intravenous, Day 1-7, Day 15-21 |
|  |  | Vincristine (VCR): 1.5 mg/m^2^, Day 1, 8, 15 |
|  |  | PEG-Asp: 2000 U/m^2^, intramuscular injection, Day 1 |
|  |  | Venetoclax (VEN): Day 1-14; three |
|  |  | Combined sheath injection (IT): Day 1, 15 |
| V | Late consolidation 1 | High-dose methotrexate: 5000 mg/m^2^ IV (first 10% infused over 30 minutes, remaining 90% infused over 23.5 hours), leucovorin, 15 mg/m^2^, orally or intravenously at 42, 48 and 54 hours after HD-MTX initiation |
|  |  | Triple intrathecal injection: Day 1, 15 |
|  |  | Thiopurine (6-MP): 25 mg/m^2^/Day, oral, Day 1-28 |
|  |  | High-dose cytarabine: 3000 mg/m^2^, IV, every 12 hours, Day 29-30 |
|  |  | PEG-Asp: 2000 U/m^2^, intramuscular injection, Day 31 |
| VI | Late consolidation-2 (CAT+) | Cyclophosphamide (CTX): 1000mg/m^2^, IV infusion, Day 1 |
|  |  | Ara-C: 50 mg/m^2^, subcutaneous/intravenous injection, every 12 hours, Day 1-7 |
|  |  | Mercaptopurine (6-MP): 40 mg/m^2^ daily, oral, Day 1-7 |
|  |  | Vincristine (VCR): 1.5 mg/m2, Day 1, 8 |
|  |  | PEG-Asp: 2000 U/m^2^, intramuscular injection, Day 1 |
|  |  | Triple intrathecal injection (IT): Day 1 |
| VII | Maintenance treatment | MTX +6-MP*, MTX +6-MP, MTX +6-MP, DEX+VCR+IT, repeated every 4 weeks for a total of 20 weeks. (6-MP: 50 mg/m^2^/day, oral, Day 1-21; MTX: 25 mg/m^2^/ day, orally, day 1, 8, 15; DEX:8mg/m^2^/day, oral, Day 22-28; VCR: 1.5 mg/m^2^, intravenous injection, Day 22; Triple intrathecal injection, Day 22) |

Note: Chemotherapy treatment according to the Chinese Children Cancer Group-Infant acute lymphoblastic leukemia-2022 collaborative group protocol (CCCG-iALL-2022).
