# Supplementary material for: Case Report: A case of infantile acute hyperleukocytic leukemia treated by leukapheresis
Source: Front Pediatr. 2024 Dec 9;12:1497943. doi: 10.3389/fped.2024.1497943 (PMC11663633; doi:10.3389/fped.2024.1497943)
Supplement: Supplementary file 2 [file Datasheet1.docx]

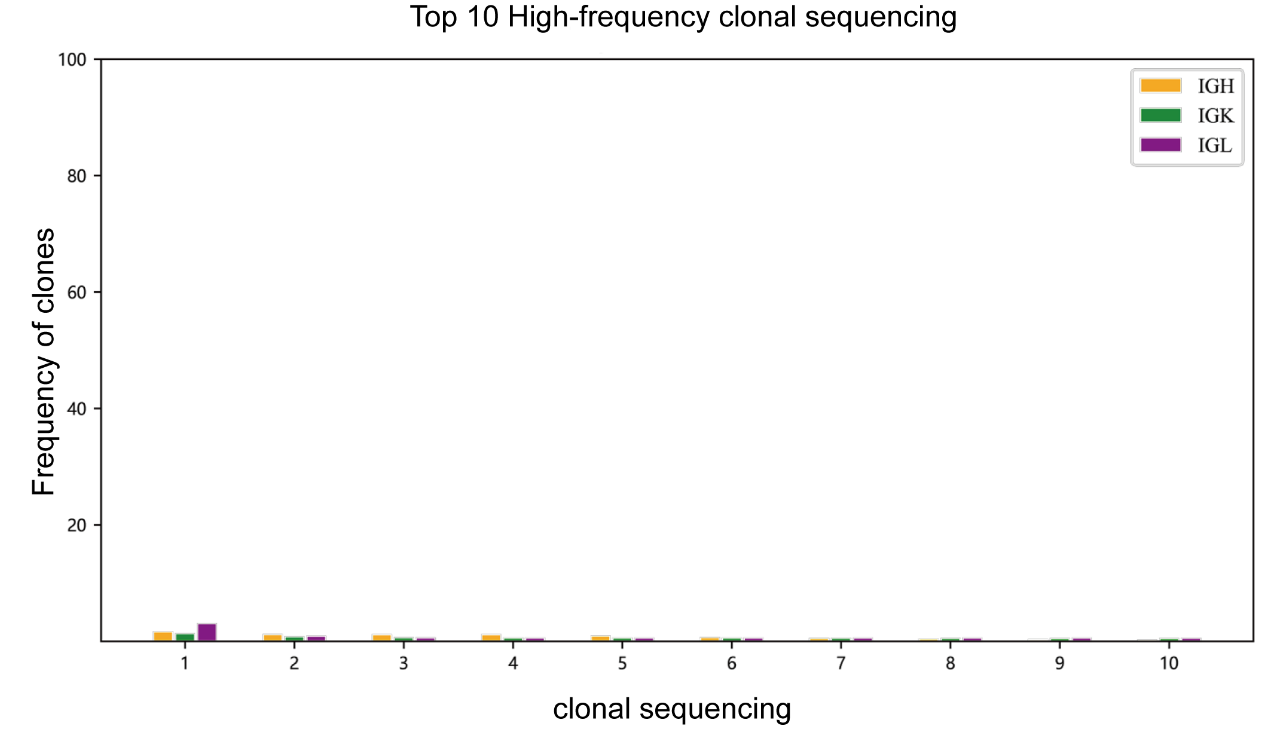


**Supplementary FIGURE 1** Results of IG rearrangement by immune repertoire sequencing using Multiplex PCR combined with next-generation sequencing technology.


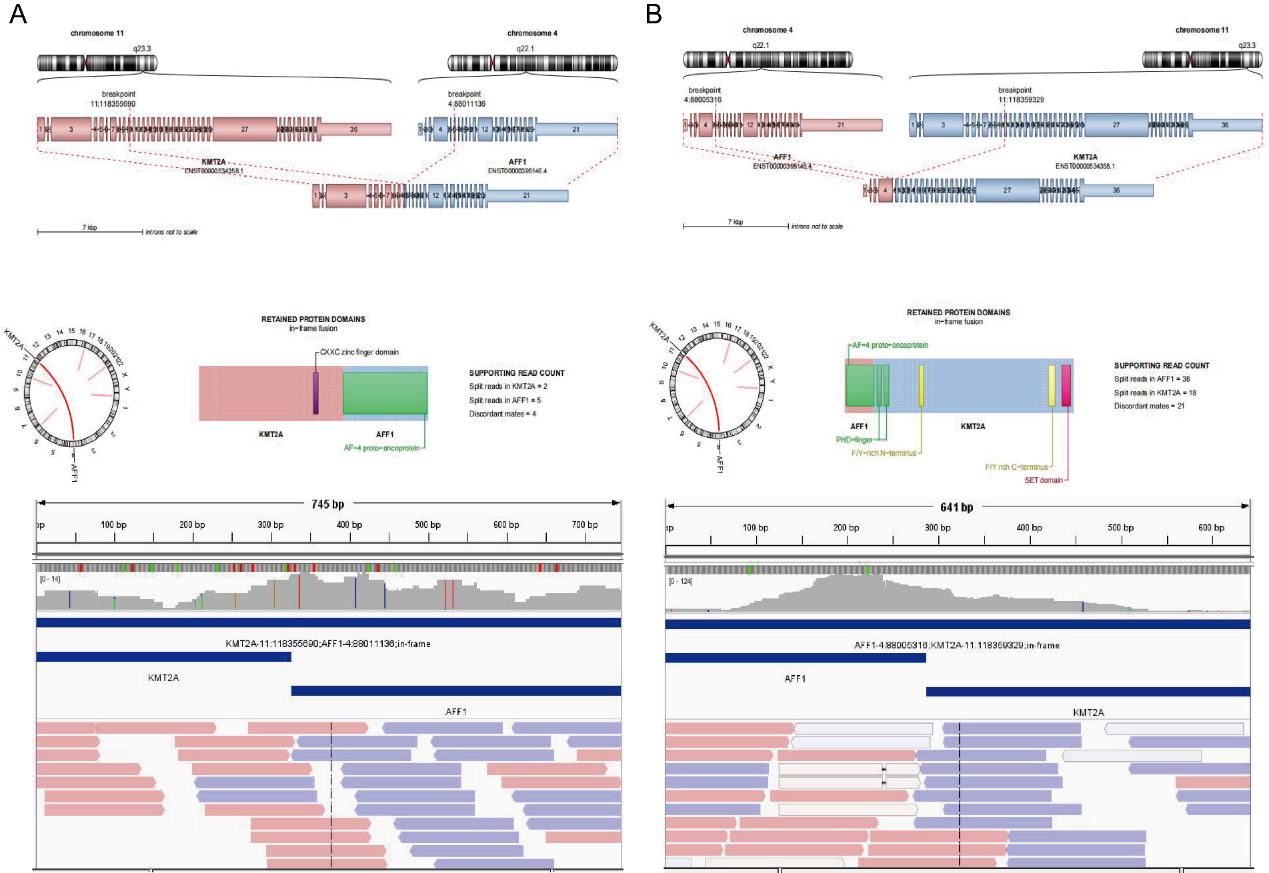


**Supplementary FIGURE 2** Principles of blood tumor transcriptome sequencing. (A) Test site from 5'-KMT2A to 3'-AFF1. (B) Test site from 5'-AFF1 to 3'-KMT2A.


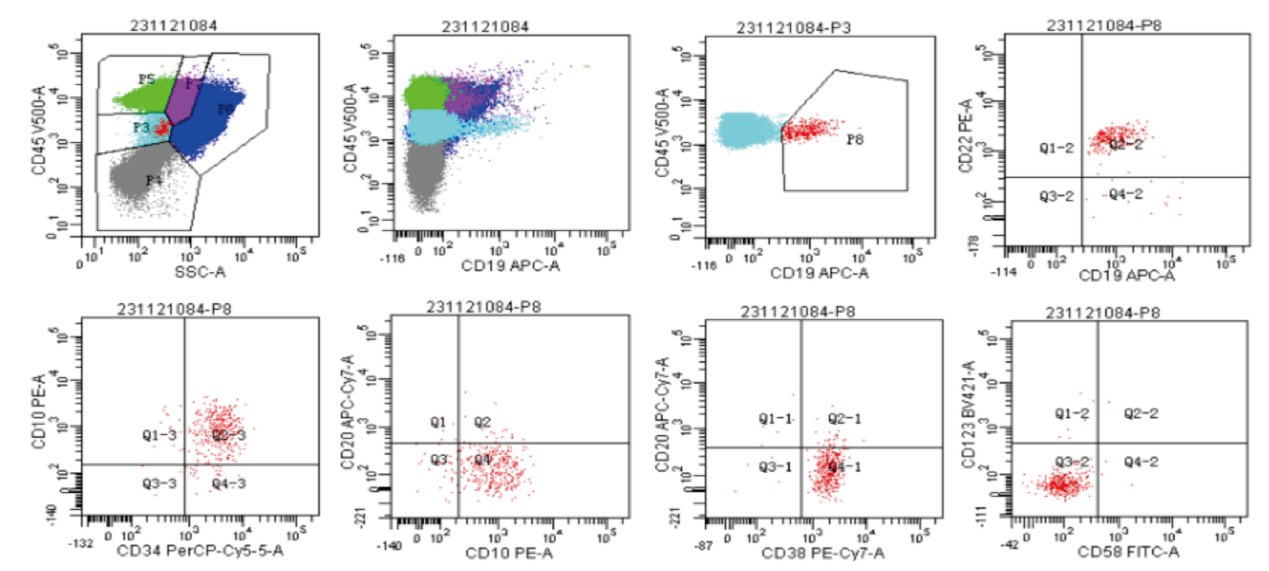


**Supplementary FIGURE 3** Detection of minimal residual disease in bone marrow tumors in the post-treatment.
